# Effect of glycemic gap on prognosis and complications in vulnerable period of acute heart failure

**DOI:** 10.5937/jomb0-52619

**Published:** 2025-03-21

**Authors:** Lin Zheng, Weifeng Zheng, Mingming Zhang, Bo Li

**Affiliations:** 1 Ningbo Fourth Hospital, Department of Cardiology, Ningbo, China

**Keywords:** glycemic gap, acute heart failure, vulnerable period of heart failure, prognosis, complication, glikemijski jaz, akutna srčana insuficijenicija, vulnerabilni period srčane insuficijencije, prognoza, komplikacija

## Abstract

**Background:**

To investigate the effect of glycemic gap on the prognosis and complications of heart failure in patients with acute heart failure.

**Methods:**

A total of 100 patients with acute heart failure admitted to our hospital from January 2022 to August 2023 were retrospectively selected, and the patients were divided into two groups according to their prognosis, the good prognosis group (n=58) and the poor prognosis group (n=42). The general data of the two groups were compared, and the influencing factors on the prognosis of patients with acute heart failure during the fragile period were analyzed by multi-factor Logistics regression. ROC analyzed the predictive value of blood sugar gap on the prognosis of acute heart failure patients during the vulnerable period of heart failure, and compared the incidence of complications between the two groups of patients..

**Results:**

The blood glucose gap, NT-proBNP, Ang II, hscTn, and SCR in patients with good prognosis were lower than those in patients with poor prognosis, and LVEF and hemoglobin were higher than those in patients with poor prognosis (P<0.05). Multivariate Logistics regression analysis results showed that blood glucose gap, NTproBNP, AngII, hs-cTn, SCR, LVEF, and hemoglobin were independent influencing factors on the prognosis of acute heart failure patients during the vulnerable period of heart failure (P<0.05), ROC analysis results showed that the area under the curve of the value of blood sugar gap in the prognosis of acute heart failure patients during the vulnerable period of heart failure was 0.6071,(95% CI: 0.3107~0.9035), Youden=0.89, at this time, the sensitiv ity was 92.8 and the specificity was 97.1. The complication rate in the good prognosis group (1.72%) was significantly lower than that in the poor prognosis group (16.67%).

**Conclusions:**

Glycemic gap is related to the prognosis and complications of heart failure in patients with acute heart failure. Hyperglycemia gap will affect the prognosis of heart failure in patients with acute heart failure, resulting in poor prognosis and increasing the incidence of complications.

## Abstract (sr)

**Uvod:**

Cilj je bio da se istraži uticaj glikemijskog jaza na prognozu i komplikacije srčane insuficijencije kod pacijenata sa akutnom srčanom insuficijencijom.

**Metode:**

Retrospektivno je odabrano ukupno 100 pacijenata sa akutnom srčanom insuficijencijom koji su primljeni u našu bolnicu od januara 2022. do avgusta 2023. godine, a pacijenti su prema prognozi podeljeni u dve grupe, sa dobrom prognozom (n=58) i lošom, grupa prognoze (n=42). Upoređeni su opšti podaci dve grupe, a faktori uticaja na prognozu pacijenata sa akutnom srčanom insuficijencijom tokom krhkog perioda analizirani su multifaktorskom logistikom regresijom. ROC je analizirao prediktivnu vrednost jaza šećera u krvi na prognozu pacijenata sa akutnom srčanom insuficijencijom tokom osetljivog perioda srčane insuficijencije i uporedio incidencu komplikacija između dve grupe pacijenata.

**Rezultati:**

Gap glukoze u krvi, NT-proBNP, Ang II, hs-cTn i SCR kod pacijenata sa dobrom prognozom bili su niži od onih kod pacijenata sa lošom prognozom, a LVEF i hemoglobin su bili vi{i od onih kod pacijenata sa lošom prognozom. (P<0,05), rezultati multivarijantne logističke regresione analize pokazali su da su jaz glukoze u krvi, NT-proBNP, AngII, hs-cTn, SCR, LVEF i hemoglobin nezavisni faktori uticaja na prognozu pacijenata sa akutnom srčanom insuficijencijom tokom ranjivog perioda srca neuspeh (P<0,05), rezultati ROC analize su pokazali da je površina ispod krive vrednosti jaza {e}era u krvi u prognozi pacijenata sa akutnom srčanom insuficijencijom tokom osetljivog perioda srčane insuficijencije 0,6071, (95% CI: 0,3107~0,9035), Iouden=0,89, u ovom trenutku, osetljivost je bila 92,8, a specifičnost 97,1. Stopa komplikacija u grupi sa dobrom prognozom (1,72%) bila je značajno niža od one u grupi sa lošom prognozom (16,67%).

**Zaključak:**

Glikemijski jaz je povezan sa prognozom i komplikacijama srčane insuficijencije kod pacijenata sa akutnom srčanom insuficijencijom. Hiperglikemijski jaz će uticati na prognozu srčane insuficijencije kod pacijenata sa akutnom srčanom insuficijencijom, što rezultira lošom prognozom i pove}anjem učestalosti komplikacija.

## Introduction

Currently, acute heart failure is a prevalent and severe cardiovascular condition that leads to inadequate organ and tissue perfusion, resulting in symptoms such as dyspnea, edema, and fatigue. This insufficiency is caused by a rapid decline in cardiac pumping function, leading to compromised organ and tissue perfusion [1]. The vulnerable period of heart failure, which refers to the early stage post-discharge, is a critical phase as indicated in the 2018 edition of Chinese guidelines for heart failure diagnosis and treatment [2]. During this period, the mortality and readmission rates of hospitalized heart failure patients within 2 to 3 months after discharge are alarmingly high at 15% and 30% respectively [3] [4]. Despite significant improvement in symptoms and signs during hospitalization, patients' cardiac function has not fully recovered, making recurrence a possibility [5] [6]. Consequently, patients in the vulnerable period of heart failure face immense risks and may require rehospitalization. Individual differences and disease severity contribute to varying prognoses [7] [8]. Patients in the vulnerable stage of acute heart failure need long-term rehabilitation and management, including lifestyle adjustments, dietary control, and regular medication, to prevent the recurrence of acute heart failure [9] [10]. Failure to address the condition promptly or with appropriate measures may lead to a bleak prognosis, exacerbating heart failure and impairing cardiac function further [6]. Glycemic gap, which measures the disparity between maximum and minimum glycemic gap concentrations throughout the day, accurately reflects glucose fluctuation in patients [11]. Several studies have demonstrated the correlation between glycemic gap and the prognosis of diseases like acute heart failure, stroke, and necrotizing fasciitis [12] [13]. However, research focusing on the vulnerable period of acute heart failure in patients is limited. Therefore, this study aims to investigate the impact of glycemic gap on the prognosis and complications of patients with acute heart failure, providing valuable clinical insights.

## Materials and methods

### Objects of study

From January 2022 to August 2023, a retrospective selection was made of a total of 100 patients who received treatment for acute heart failure at our hospital. The patients were chosen based on specific inclusion and exclusion criteria. The inclusion criteria were as follows: (1) patients who met the clinical diagnosis of acute heart failure and were in the vulnerable stage of heart failure, as outlined in the guidelines for Emergency Management of Acute Heart failure (2022) [14]; (2) patients aged 18 years or older; and (3) patients with complete clinical data available. Exclusion criteria were as follows: (1) patients with malignant tumors; (2) patients with mental disorders; (3) patients who had received corticosteroid treatment in the past three months; and (4) patients with infectious diseases. Prior approval from the Ethics Committee was obtained, and all patients or their families provided signed informed consent forms.

### Methods

The electronic medical record system was utilized to collect the general patient data. In accordance with the guidelines outlined in the Chinese Emergency Management of Acute Heart failure (2022), the patients were categorized into two groups based on their prognosis: a good prognosis group (consisting of 58 patients) and a poor prognosis group (comprising 42 patients). Prognostic criteria: good prognosis: NYHA cardiac function grade I∼II, 6-minute walk test > 450 m, and there was no readmission or cardiogenic death within 90 days after discharge.

The prognosis was poor: patients with NYHA cardiac function grade III∼IV, 6-minute walking distance less than 450 m, or patients with recurrent heart failure or cardiac death within 90 days after discharge.

Blood sugar was monitored at 7 time points throughout the day, namely before three meals, 2 hours after three meals, before going to bed or at night.

Measurement with a blood glucose meter: Use a calibrated blood glucose meter to measure blood glucose at each selected time point. Make sure to follow the meter's instructions for use correctly to obtain accurate blood sugar values.

Record blood sugar data: Record the blood sugar value of each measurement, including the measurement time, blood sugar value, and possible influencing factors such as diet and exercise. Blood glucose notebooks were used to record them for subsequent analysis and summary.

Blood sugar gap: Calculation method: Find the maximum and minimum of all blood sugar measurements during the day, and then calculate the difference. Reflects the maximum fluctuation range of blood sugar during the day.

### Statistical analysis

The analysis in this study was conducted using Statistic Package for Social Science (SPSS) 27.0 (IBM, Armonk, NY, USA). When the measurement data followed a normal distribution, they were represented as x̄ ± s. The comparison between groups of measurement data was performed using t-tests. The counting data, on the other hand, were expressed as »n%« and the comparison was carried out using the χ^2^ test or the Fisher exact method.

## Results

### Univariate analysis of prognostic factors in vulnerable period of acute heart failure

There were no noticeable disparities in age, gender, hypertension history, BMI, TG, diabetes history, and serum potassium levels between the two groups. However, the good prognosis group exhibited lower levels of glycemic gap, BNP, Ang II, cTnI, and SCR compared to the poor prognosis group. Conversely, the good prognosis group had higher levels of LVEF and hemoglobin compared to the poor prognosis group (Table 1).

**Table 1:** Comparison of general information.

General information	good prognosis group<br>(n=58)	poor prognosis group<br>(n=42)	t/χ^2^	P
Age (years)	70.86±4.54	71.67±4.52	1.296	<0.198
Gender male (female)	28 (30)	21 (21)	0.029	<0.986
Hypertension is (No)	21 (37)	16 (26)	0.037	<0.982
BMI (kg/m^2^)	25.16±1.20	25.51±1.28	1.400	<0.165
TG (mmol/L)	1.49±0.60	1.56±0.47	0.629	<0.531
Diabetes is (No)	16 (42)	9 (33)	0.493	<0.782
Serum potassium (mmol/L)	4.00±0.54	4.11±0.51	1.029	<0.306
Glycemic gap	0.96±0.34	2.34±0.65	13.789	<0.000
BNP (pg/mL)	505.62±14.92	516.05±15.50	3.394	<0.001
Ang II (ng/L)	30.55±3.65	42.07±2.87	16.993	<0.000
LVEF (%)	53.26±4.23	50.22±4.02	3.621	<0.000
cTnI (ng/mL)	0.56±0.02	0.64±0.01	23.832	<0.000
SCR (mmol/L)	98.79±21.36	111.61±19.54	3.069	<0.003
Hemoglobin (gmax/L)	142.19±9.35	134.15±10.71	3.992	<0.000

### Multivariate logistics regression analysis of prognostic factors in vulnerable phase of heart failure in patients with acute heart failure

In the analysis of acute heart failure patients, various independent variables such as the difference in glycemic gap, BNP, Ang II, cTnI, SCR, LVEF, and hemoglobin were considered. The dependent variable was the prognosis, categorized as poor prognosis (1) or good prognosis (0). Through Binary Logistics regression analysis, it was found that the aforementioned factors, including glycemic gap difference, BNP, Ang II, cTnI, SCR, LVEF, and hemoglobin, independently influenced the prognosis of patients with acute heart failure (Table 2).

**Table 2:** Multivariate Logistics regression analysis of prognostic factors in vulnerable phase of acute heart failure.

Factors	β	SE	Ward	OR	95%CI	*P*
Glycemic gap	0.748	0.643	12.453	2.113	0.599~7.451	<0.001
BNP (pg/mL)	1.142	0.345	10.951	3.132	1.593~6.159	<0.001
Ang II (ng/L)	0.622	0.254	5.990	1.862	1.132~3.063	<0.001
LVEF (%)	0.747	0.304	5.430	2.111	1.163~3.831	<0.001
cTnI (ng/L)	0.817	0.349	5.482	2.264	1.142~4.487	<0.001
SCR (mmol/L)	0.685	0.294	5.430	1.984	1.115~3.530	<0.001
Hemoglobin (g/L)	0.758	0.324	5.480	2.135	1.131~4.029	<0.001

### The value of ROC analysis of glycemic gap in predicting the prognosis of patients with acute heart failure in vulnerable period

**Figure 1:**
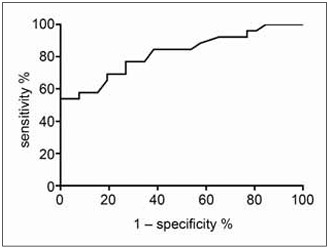
The value of ROC analysis of glycemic gap in predicting the prognosis of patients with acute heart failure in vulnerable period.

In the vulnerable period of acute heart failure patients, the prognosis can be effectively assessed using ROC analysis. The area under the glycemic gap value curve was determined to be 0.8071. The sensitivity of the assessment was 72.8, while the specificity was 70.5, as illustrated in Figure 1.

### Comparison of complications between the two groups

The incidence of complications in the good prognosis group (1.72%) was significantly lower than that in the poor prognosis group (16.67%), as shown in Table 3.

**Table 3:** Comparison of complications between the two groups.

Grouping	Number of cases	Arrhythmia	angina	Cardiogenic shock	blood clots	Incidence (%)
Good prognosis group	58	1	0	0	0	1.72
Poor prognosis group	42	3	2	1	1	16.67
*χ^2^*						7.390
*P*						0.025

## Discussion

During the critical phase of acute heart failure, patients often experience unfavorable outcomes and complications. Previous research has indicated that the disparity in glycemic gap is a reliable predictor of prognosis for individuals with acute myocardial infarction [15]. Nevertheless, limited investigations have explored the potential associations between prognosis, complications, and the blood glucose gap in patients with acute heart failure during this vulnerable period [16] [17]. Hence, this study aims to investigate the correlation between the glycemic gap and the prognosis, as well as the occurrence of complications, in patients with acute heart failure.

The findings from the logistic regression analysis using a binary model indicated that the glycemic gap was an independent variable influencing the prognosis of patients with acute heart failure during the vulnerable period. The ROC analysis demonstrated that the area under the curve (AUC) for the glycemic gap in predicting the prognosis of these patients was 0.6071, (95%CI: 0.3107~0.9035), yielding a Youden index of 0.89. The sensitivity and specificity were 92.8 and 97.1, respectively. The results of Liao WI et al. [17] also showed that blood sugar gaps can predict in-hospital mortality in acute psychological failure. The results showed that patients with blood sugar gaps> 43 mg/dL had a higher all-cause mortality rate (adjusted hazard ratio 7.225, 95% confidence interval 1.355-38). 520) Patients with blood glucose gap levels 43 mg/dL,These results suggest that the glycemic gap shows promising predictive value for the prognosis of patients with acute heart failure during the vulnerable period, with lower glycemic gap associated with better outcomes. High glycemic gap, as indicated by the glycemic gap, are associated with a poor prognosis in these patients [18]. Increased glycemic gap lead to metabolic disturbances, heightened inflammatory response, and oxidative stress [19] [20]. These metabolic disturbances impact energy metabolism, affecting the normal systolic and diastolic function of the myocardium. The inflammatory response exacerbates the symptoms and progression of heart failure, negatively impacting patient prognosis [21]. Oxidative stress further damages cardiomyocytes, compromising myocardial function and worsening heart failure symptoms [22] [23]. The analysis of complications in the two patient groups revealed a significantly lower incidence of complications in the group with a favorable prognosis (1.72%) compared to the group with a poor prognosis (16.67%). This suggests that lower glycemic gap can reduce the risk of complications. The reason may be that patients with lower glycemic gap exhibit better metabolic profiles, which provide cardiovascular protection through mechanisms such as improved endothelial function and reduced inflammatory response [24] [25].

Furthermore, the findings also indicated that patients with a favorable outlook exhibited lower levels of BNP, Ang II, cTnI, and SCR, compared to those with a less favorable prognosis. Conversely, patients with a positive prognosis demonstrated higher levels of LVEF and hemoglobin, in contrast to those with a poorer prognosis. BNP, Ang II, cTnI, SCR, LVEF, and hemoglobin also emerged as independent prognostic factors for acute heart failure patients [26]. These parameters are crucial indicators that reflect the severity and prognosis of heart failure [27], and their fluctuations can signify changes in heart failure among patients. The outcomes further emphasize that patients with a favorable prognosis have superior cardiac function and enhanced blood oxygenation [28] [29]. In research to investigate the impact of blood sugar gaps on the prognosis and complications of acute heart failure (AHF) patients during the vulnerable period of heart failure, although some important findings have been made, there are still some limitations. First, the study sample size is relatively limited, which may affect the broad applicability of the results. A larger sample size can more accurately reflect the relationship between blood sugar gaps and heart failure prognosis. Second, the study's follow-up time may not be sufficient to fully assess long-term outcomes. The prognosis of heart failure is often long-term, and short-term follow-up may not fully reveal the impact of blood sugar gaps on long-term prognosis.

In conclusion, there exists a correlation between the discrepancy in glycemic gap and the prognosis and complications of acute heart failure in patients. Elevated blood sugar ranges may exacerbate the symptoms and progression of heart failure, resulting in a bleak prognosis and a higher occurrence of complications. Consequently, it is imperative to closely monitor blood sugar fluctuations and enhance prognosis and quality of life by regulating blood sugar levels and minimizing the variance in blood sugar. Additionally, for individuals with hyperglycemia, it is crucial to strengthen monitoring and implement intervention strategies in order to diminish the likelihood of complications.

## Dodatak

### Conflict of interest statement

All the authors declare that they have no conflict of interest in this work.
